# Tracheobronchial Foreign Bodies in Children: Experience From 1,328 Patients in China

**DOI:** 10.3389/fped.2022.873182

**Published:** 2022-05-26

**Authors:** Ling Ding, Shuping Su, Cheng Chen, Hongbing Yao, Ling Xiao

**Affiliations:** ^1^Department of Otolaryngology-Head and Neck Surgery, Children's Hospital of Chongqing Medical University, Chongqing, China; ^2^National Clinical Research Center for Child Health and Disorders, Chongqing, China; ^3^Ministry of Education Key Laboratory of Child Development and Disorders, Chongqing, China; ^4^Chongqing Key Laboratory of Pediatrics, Chongqing, China; ^5^Chongqing Higher Institution Engineering Research Center of Children's Medical Big Data Intelligent Application, Chongqing, China

**Keywords:** foreign body, aspiration, tracheobronchial, children, bronchoscopy

## Abstract

**Objective:**

To analyze the clinical characteristics of tracheobronchial foreign body (FB) cases in a pediatric Chinese population.

**Methods:**

The clinical data of pediatric patients aged 0–18 years old diagnosed with a tracheobronchial FB in the Children's Hospital of Chongqing Medical University between September 2018 and August 2021 were analyzed retrospectively.

**Results:**

Among 1,328 included cases, 92.09% of patients were <3 years old, the male to female ratio was 1.86:1. The prevalence of tracheobronchial FB was similar between patients living in rural and urban areas and tracheobronchial FBs were more common in winter. The most common presenting symptoms were cough and wheezing. The most common CT findings was local obstruction or tracheobronchial narrowing, followed by obstructive emphysema of lung and pneumonia. The 11.3% of cases that did not report FB aspiration on admission had a longer time to hospital admission and longer hospitalization time than cases reporting FB aspiration (*P* < 0.05). The most common FB type was nuts (81.17%). FBs were more frequently located in the right bronchus, and 64 (4.82%) cases involved multiple FBs. FBs were expelled by coughing in only 4.07% of cases. For the other cases, FB removal by first bronchoscopy in our hospital was successful and complete in 96.86% of cases. 1.51% of patients had hypoxic ischemic encephalopathy (HIE) and the location of FBs was a possible risk factor for HIE (*P* < 0.05).

**Conclusions:**

Tracheobronchial FBs occurred most commonly in children <3 years old. If asphyxia occurs in children which FBs aspirated, emergency treatment is needed to reduce the occurrence of HIE. Rigid bronchoscopy remains the first-line method for FB removal in children.

## Introduction

Tracheobronchial foreign body (FB) is one of the most common acute and severe diseases in children. Previous research has shown that tracheobronchial FB accounts for 7.9–18.1% of accidental injuries in children aged 0–14 years in China and 80% in children aged 1–3 years (1). The symptoms of tracheobronchial FB vary depending on the size of the FB, the grade of obstruction, and the location of the FB. Patients can present with coughing, wheezing, stridor, fever or even dyspnea, while a few patients are almost asymptomatic. Tracheobronchial FB can cause many complications, some serious patients may experience cardiopulmonary arrest and sudden death (2, 3). Fortunately, early diagnosis and surgical treatment can effectively reduce the complications of tracheobronchial FBs as well as the mortality among children (4, 5). However, misdiagnosis, missed diagnosis, surgical failure, and serious complications occur due to various reasons in clinical practice, bringing great harm and burden to children and their families. Therefore, timely diagnosis and treatment of tracheobronchial FBs are very urgent.

To investigate the latest epidemiological and clinical characteristics of pediatric patients with tracheobronchial FBs, the case data from the Children's Hospital of Chongqing Medical University were retrospectively collected. The hospital is the third largest children's hospital in China and provides services mainly for children in southwest China. Data for all cases treated over 3 years were collected to analyze the patients' general situation, disease characteristics, clinical symptoms, imaging examinations, as well as the FB type, location, treatment and complications.

## Materials and Methods

### Study Design and Data Collection

We retrospectively studied all pediatric cases younger than 18 years diagnosed as tracheobronchial FB in the Children's Hospital of Chongqing Medical University between September 2018 and August 2021. As a retrospective study, no consent was required for the analysis of the data collected from medical records. However, written informed consent was given by patients or their guardians before bronchoscopy. The research protocol was approved by the Medical Ethics Committee of Children's Hospital of Chongqing Medical University.

Cases were suspected according to a report of FB aspiration by the patient or their guardian, clinical symptoms, physical examination findings, or imaging examination findings. The diagnosis of FB was confirmed by one of two ways: (1) the patient coughed up a FB. If after coughing, no FBs were found on subsequent imaging examination, and the patient's symptoms and physical signs were resolved, the FB was considered to have been eliminated by coughing; or (2) a FB was observed by bronchoscopy.

### Study Methods and Statistical Analysis

The extracted patient data included age, gender, residential area (urban or rural), report of FB aspiration, clinical symptoms (e.g., cough, wheezing/laryngeal stridor/laryngeal noise, fever, dyspnea), imaging examination findings before surgery [chest computed tomography (CT)], endoscopic findings (e.g., type of FB, FB location, bronchoscopy type, time of operation), complications related to FBs, and time from aspiration to hospital admission as well as hospitalization time.

The collected data were processed and analyzed using SPSS 23 statistical software (IBM, Armonk, NY, USA). If the measurement data were in line with normal distribution, *t*-test was used for comparisons between two groups; if not, Mann-Whitney U test was used for comparisons between two groups. For categorical data, Chi-square test, Continuous calibration chi-square test or Fisher's exact test was used for univariate analysis. Differences for which *P* < 0.05 were considered statistically significant for all analyses.

## Results

### Patients' Characteristics and Symptoms

A total of 1,328 patients diagnosed with tracheobronchial FBs were included in the present study. The male to female ratio of patients was 1.86:1. The age of the patients ranged from 4 months to 13 years, and the median age was 1.54 years. The distributions of cases according to gender, age group, residence type, seasonal occurrence, symptoms, and report of FB aspiration are presented in Table 1.

**Table 1:** Patient characteristics and symptoms (*n* = 1,328).

**Variable**	***n***	**%**
**Sex**
Male	864	65.06
Female	464	34.94
**Age**
0–1 years	132	9.94
1–2 years	895	67.39
2–3 years	196	14.76
3–6 years	58	4.37
≥6 years	47	3.54
**Residence type**
Urban	712	53.61
Rural	616	46.39
**Season**
Spring	335	25.23
Summer	196	14.76
Autumn	368	27.71
Winter	429	32.30
**Symptoms**
Cough	1,303	98.12
Wheezing/laryngeal stridor/laryngeal noise	1,242	93.52
Fever	369	27.79
Dyspnea	92	6.93
**Report of FB aspiration**
Yes	1,178	88.70
No	150	11.30

### Time Before Hospital Admission and Hospitalization Time

Time before hospital admission was defined as the duration of time from when the aspiration event occurred, if witnessed, or from the onset of symptoms to when the patient was admitted at our hospital. The time before hospital admission ranged from 1 h to 2 years. The shortest hospitalization time was only 1 h in a case in which the patient coughed up the FB after admission, whereas the longest hospitalization time was 34.4 days. Table 2 compares the time before hospital admission and the hospitalization time according to whether the patient or guardian reported aspiration of a FB. The differences in both times were statistically significant (*P* < 0.001).

**Table 2:** Influence of reporting of FB aspiration on the time before hospital admission and hospitalization time.

	**No report of FB**	**Report of FB**
**Variable**	**aspiration (*n* = 150)**	**aspiration (*n* = 1,178)**	***P-*value**
Time before	20.00 (8.75, 31.00)	3.00 (1.00, 10.00)	<0.001
hospital
admission,
days
Hospitalization	4.71 (3.36, 6.76)	3.24 (2.38, 4.16)	<0.001
time, days

*Values are presented as median (first quartile - third quartile). Mann-Whitney U test was used to test statistical difference*.

### CT Findings

Table 3 shows the CT findings of FBs. The most frequent CT finding was local obstruction or tracheobronchial narrowing because of FBs (99.17%), followed by obstructive emphysema of lung in 990 cases (74.55%) and pneumonia in 516 cases (38.86%).

**Table 3:** CT findings of FBs (*n* = 1,328).

**CT findings**	***n***	**%**
Local obstruction or tracheobronchial	1,317	99.17
narrowing because of FBs
Obstructive emphysema of lung	990	74.55
Pneumonia	516	38.86
Atelectasis	149	11.22
Pneumothorax, subcutaneous emphysema	45	3.39
and mediastinal emphysema
Laryngeal edema and laryngeal	12	0.90
obstruction
Pleural effusion	6	0.45

### Locations of FBs

The distribution of FBs found at different locations are presented in Table 4. Among all cases, the highest percentage of FBs were located in the right main bronchus (38.33%). Additionally, more FBs were located in the right bronchus than in the left bronchus (49.70 vs. 38.63%). In 64 cases (4.82%), multiple FBs were identified.

**Table 4:** Locations of FBs (*n* = 1,328).

**FB location**	***n***	**%**
Trachea	91	6.85
Right main bronchus	509	38.33
Right upper bronchus	8	0.60
Right middle bronchus	68	5.12
Right lower bronchus	75	5.65
Left main bronchus	391	29.44
Left upper bronchus	22	1.66
Left lower bronchus	100	7.53
Multiple FBs	64	4.82

### Types of FBs

The majority (93.52%) of FBs found in these children were organic objects, consisting of mostly food items. Nuts accounted for 81.17% of the FBs, with peanuts, seeds, and walnuts found most often. Only 5.42% of the FBs were inorganic objects, with pen point or cap accounting for 1.96%, followed by plastic paper/film and plastic toys (Table 5).

**Table 5:** Types of FBs (*n* = 1,328).

**Types of FBs**	***n***	**%**
Organic objects	1,242	93.52
Peanut	573	43.15
Seed	201	15.14
Walnut	108	8.13
Bean	70	5.27
Chinese chestnut	30	2.26
Other nuts	96	7.23
Fruit	48	3.61
Bone or shrimp shell	47	3.54
Meat or animal product	8	0.60
Corn	7	0.53
Other food or residue	43	3.24
Other organic object	11	0.83
Inorganic objects	72	5.42
Pen point or cap	26	1.96
Plastic toy	10	0.75
Plastic paper	10	0.75
Light diode	4	0.30
Metal screw or nut	4	0.30
Tin or aluminum foil	4	0.30
Thumb tack	3	0.23
Whistle	3	0.23
Pegwood	2	0.15
Stone	2	0.15
Metal buckle	2	0.15
Needle	1	0.08
Magnet	1	0.08
Undetermined	14	1.05

### Treatments

Of the 1,328 cases examined, in 54 (4.07%) cases the patient was able to cough up the FB by themselves. The other cases were removed FBs only underwent rigid bronchoscopy and/or flexible bronchoscopy. The surgeons of department of Thoracic Surgery and Otorhinolaryngology used holding forceps, grasping forceps or cup-shaped forceps to remove FBs underwent rigid bronchoscopy. While respiratory physicians used mainly basket-shaped forceps and tooth forceps, and few cases cooperated with cryoprobe, balloon catheter and other methods.

Table 6 summarizes the details of the endoscopic removal of FBs in the other 1,274 cases. FBs were removed during the first bronchoscopy in 1,215 (95.37%) cases, during the second bronchoscopy in 52 (4.08%) cases, during a third bronchoscopy in 6 cases, and during a fourth bronchoscopy in 1 child. In a few cases, complete removal of FBs had not been achieved in other hospitals, and the children were then transferred to our hospital. Among the cases who received first surgical treatment in our hospital, 96.86% of cases were removed FBs successfully and completely underwent first bronchoscopy.

**Table 6:** Endoscopic removal of FBs (*n* = 1,274).

**Endoscopic removal**	***n***	**%**
One bronchoscopy procedure	1,215	95.37
One rigid bronchoscopy^a^	987	77.47
One flexible bronchoscopy^a^	228	17.90
Two bronchoscopy procedures^a^	34	2.67
One bronchoscopy procedure in	18	1.41
another hospital and one in our hospital
Three bronchoscopy procedures^b^	6	0.47
Four bronchoscopy procedures^c^	1	0.08

a *Bronchoscopy in our hospital*.

b *More than one procedure in our hospital*.

c *Three procedures in another hospital and one in our hospital*.

### A Severe Complication- HIE

No case died related to endoscopic procedure and FBs in all included cases, so we analyzed the clinical characteristics of hypoxic ischemic encephalopathy (HIE) which was also a serious complication in the present study. There were 20 children (1.51%) with HIE. They were all caused by hypoxia because of the airway obstruction related to FBs before bronchoscopy. Then the study analyzed clinical characteristics and divided all the children into two groups: with HIE, without HIE (Table 7). 6.59% of cases with tracheal FBs and 4.69% of cases with multiple FBs were associated with HIE, which was significantly more than bronchial FBs. And the time before hospital admission was statistically different between the two groups (*P* < 0.05).

**Table 7:** Risk factors of HIE (*n* = 1,328).

**Variable**	**Without HIE (*n* = 1,308)**	**With HIE (*n* = 20)**	***P-*value**
**Sex**			0.641^a^
Male	850	14	
Female	458	6	
Age, years	1.55 (1.24, 1.95)^b^	1.42 (0.88, 2.11)^b^	0.346^c^
Residence type			0.900^a^
Urban	701	11	
Rural	607	9	
Report of FB aspiration			0.589^d^
Yes	1,159	19	
No	149	1	
Time before hospital admission, days	4.00 (1.00, 12.00)^b^	0.75 (0.17, 4.50)^b^	0.001^c^
Hospitalization time, days	3.34 (2.44, 4.47)^b^	3.50 (2.81, 8.40)^b^	0.090^c^
Type of FB^e^			1.000^d^
Organic objects	1,223	19	
Inorganic objects	71	1	
FB location			<0.001^f^
Trachea	85	6	
Right bronchus	655	5	
Left bronchus	507	6	
Multiple FBs	61	3	

a *Chi-square test was used*.

b *Values are presented as median (first quartile - third quartile)*.

c *Mann-Whitney U test was used*.

d *Continuous calibration chi-square test was used*.

e *n = 1,314, because types of FB in14 cases were undetermined*.

f *Fisher's exact test was used*.

## Discussion

This retrospective analysis of all pediatric tracheobronchial FB cases treated in the Children's Hospital of Chongqing Medical University between September 2018 and August 2021 identified several trends in the characteristics of these cases. In the present study, the male-to-female ratio among the cases was 1.86:1, which is similar to the 2:1 ratio reported in several previous studies (6–10) and slightly less than the ratio of 2.6:1 reported by another study (11). However, several studies have reported similar incidence rates of tracheobronchial FBs in both genders (12–14). Thus, more large-scale epidemiological studies are needed to confirm whether tracheobronchial FBs are more prevalent in boys than in girls.

Age was an important factor in tracheobronchial FB cases, with 92.09% of cases in the present study occurring in children 3 years of age or less, with the peak incidence of 67.39% occurring in children 1-2 years old, a finding that is consistent with the age trends reported by most previous studies (1, 8, 10–12). Surprisingly, in three cases in the present study, the children <6 months of age, which is younger than even the youngest cases reported in most previous studies (11, 12, 15). Accordingly, clinicians should be aware of a possible recent trend of decreasing age among tracheobronchial FB cases. Because the fatality rate for tracheobronchial FBs in children <1 year old is reportedly as high as 40% (16), it is important that careful attention is given to preventing FB aspiration by children <1 year old in order to reduce accidental death.

We observed similar incidence rates of tracheobronchial FB between patients who lived in rural and urban areas, which is different from the results of a previous study that found a higher incidence of FB diagnoses in rural areas in China (1). A possible explanation is that the data were collected from a single-center located in an urban area, and some cases of FBs among patients who live in rural areas may have been treated in hospitals more local to them, while children living in the urban area near our hospital were more likely to be brought to our hospital. Another contributing factor could be that among families in urban areas, it is more common for both parents to work full-time while children are watched by elderly caretakers. Cases of tracheobronchial FBs were more common in winter, followed by autumn and spring, and least common in summer, which is consistent with the findings of a previous study in China (1). A main reason is related to the Spring Festival in China and the cold weather, which leads to the consumption of more nuts, while fewer nuts are eaten during the hot weather of the summer months.

The most common symptoms of tracheobronchial FBs were cough (98.12%) and wheezing/laryngeal stridor/laryngeal noise (93.52%), in agreement with the findings of previous studies (1, 11, 17, 18). In the present study, 11.3% of the children had no definite or suspected recent history of FB aspiration. Compared with the cases for which aspiration was reported, the children for whom aspiration was not reported had both a longer time to hospital admission and a longer hospitalization time (both *P* < 0.05). Zhou et al. (19) also confirmed that FB aspiration reporting can affect the time at which children receive medical treatment, and two other studies confirmed the hospitalization time is longer when FB aspiration is not witnessed vs. when it is (15, 20). When FB aspiration is not reported by the patient or their guardian, timely bronchoscopy is indicated if suggestive symptoms and/or suggestive physical and/or radiological findings are observed (21). Thus, may reduce the time before surgery and the hospitalization time.

The most common CT finding of tracheobronchial FBs was local obstruction or tracheobronchial narrowing (99.17%), which is similar to the previous studies (1, 22). The second most common CT finding was obstructive emphysema of lung (74.55%). So we should be alert to tracheobronchial FB when imaging examination showed obstructive emphysema. The incidence of pneumonia was 38.86% which was higher than many other previous retrospective studies (11, 18, 23). The possible explanation maybe the imaging examination finding were found only by CT in our study. Whereas, other studies used Chest X-Ray with or without CT to scan FBs. CT scan shows greater diagnostic sensitivity and specificity than Chest X-Ray (1, 24).

In the present study, the most common location of FBs was the right main bronchus (38.33%). Because the right bronchus is straighter and has larger diameter than the left, entrapment of FBs is more likely in this bronchus (25), and our finding is consistent with the results of most retrospective studies previously conducted (6, 9, 17, 18, 26, 27), but inconsistent with the findings of a few studies (8, 11). Given the observation of multiple FBs in 64 cases (4.82%) in the present study, it is important to explore the complete bronchial tree systematically when searching for FBs.

In only 4.07% of cases in the present study were children able to cough up FBs by themselves. When the symptoms and physical signs of children who are highly suspected of having a tracheobronchial FB are obviously relieved or expulsion of a FB is witnessed, it is necessary to perform a timely chest imaging examination to avoid the unnecessary use of general anesthesia for bronchoscopy. At present, the main treatment methods for tracheobronchial FB removal are rigid bronchoscopy and flexible bronchoscopy. If such treatment is unsuccessful or a FB is particularly difficult to remove, tracheotomy and thoracic surgery can be used to remove FBs. In recent years, because some centers lack the equipment or training to perform rigid bronchoscopy and training courses for flexible bronchoscopy are more widely available, flexible bronchoscopy has been increasingly used to remove FBs (28). Still, rigid bronchoscopy is the most important method used for tracheobronchial FBs removal in children, because it can provide good ventilation during the procedure and has a success rate of essentially 100% when performed by an experienced physician (29). In the present study, most of the FBs were removed during the first rigid bronchoscopy procedure. Flexible bronchoscopy was first preferred for only a few patients with FBs that were difficult to remove because of their location in the upper lobe of the bronchus or segmental bronchus or for patients treated in a newly established hospital without the capability for rigid bronchoscopy. The success rate of first bronchoscopy in the present study was 96.86%, which was similar to previously reported rates (1, 8).

Because of the disagree of guardians and protection the children from radiation, many cases lacked imaging examinations after endoscopic procedure. And different clinicians have subjective differences in the diagnosis of complications. These reasons lead to the diagnosis of complications related to endoscopic procedure and FBs removal is not accurate, so we were unable to well analyze all the complications in hospitalization. But we analyzed the clinical characteristics of HIE which could be diagnosed accurately in hospitalization. As a severe complication, the incidence of HIE was 1.51% (20 cases) in the present study which less than the ratio (2.2%) reported by Kim et al. (30). HIE can cause long-term damage, greatly affecting the children and their families. The study shows that location of FBs in trachea and multiple FBs is a risk factor for HIE. The reason maybe that the trachea FBs are more easy to cause complete obstruction by affecting ventilation of both lungs than unilateral bronchial FBs (31). And multiple FBs are more likely to lead more obstruction of airway than unilateral bronchial FBs. In addition, children with asphyxia are need emergency treatment, so the time before hospital admission of HIE group was short than without HIE group. But the number of HIE cases was small in the present study. More studies are needed to explore the risk factors for the HIE due to tracheobronchial FBs.

This study has some limitations. It was just a single-center study in one hospital and a retrospective study. Then we haven't analyzed all the complications in hospitalization because of some reasons. At last, because the large number of cases, and some children did not go to medical institutions for follow-up after leaving the hospital, we gave up the follow-up of long-term complications. But our study has a large number with 1,328 children and analyzes the clinical characteristics of tracheobronchial FBs in southwest China. More important, our study reported the incidence of FBs which were expelled by coughing and the incidence of HIE. And we found cases that did not report FB aspiration on admission had a longer time to hospital admission and longer hospitalization time than cases reporting FB aspiration. We also found the risk factors for HIE which there is almost no previous study analyzed.

## Conclusion

The suspected diagnosis of a FB is mainly based on a reported history of aspiration, respiratory symptoms, physical and imaging examination results. Therefore, when a child under 3 years presents with coughing and wheezing, it is important to inquire about any possible FB aspiration. Imaging examination is necessary when clinical symptoms and physical examinations change before surgery to avoid unnecessary bronchoscopy after a FB has been expelled by coughing. During surgery, careful exploration of the bronchial tree is needed to ensure any FBs have been completely removed. HIE is a serious complication of tracheobronchial FBs, and emergency treatment is needed to reduce the occurrence of HIE when asphyxia occurs in children which FBs aspirated.

## Data Availability Statement

The raw data supporting the conclusions of this article will be made available by the authors, without undue reservation.

## Ethics Statement

The studies involving human participants were reviewed and approved by the Medical Ethics Committee of Children's Hospital of Chongqing Medical University. Written informed consent to participate in this study was provided by the participants' legal guardian/next of kin.

## Author Contributions

LD collected data and wrote the paper. SS finished statistical analysis. CC and HY continued to check data and paper. LX proposed ideas and finished project administration. All authors have read and approved the final manuscript.

## Conflict of Interest

The authors declare that the research was conducted in the absence of any commercial or financial relationships that could be construed as a potential conflict of interest.

## Publisher's Note

All claims expressed in this article are solely those of the authors and do not necessarily represent those of their affiliated organizations, or those of the publisher, the editors and the reviewers. Any product that may be evaluated in this article, or claim that may be made by its manufacturer, is not guaranteed or endorsed by the publisher.
